# Functional disorganization of small-world brain networks in mild Alzheimer's Disease and amnestic Mild Cognitive Impairment: an EEG study using Relative Wavelet Entropy (RWE)

**DOI:** 10.3389/fnagi.2014.00224

**Published:** 2014-08-26

**Authors:** Christos A. Frantzidis, Ana B. Vivas, Anthoula Tsolaki, Manousos A. Klados, Magda Tsolaki, Panagiotis D. Bamidis

**Affiliations:** ^1^Laboratory of Medical Physics, Faculty of Health Sciences, Medical School, Aristotle University of ThessalonikiThessaloniki, Greece; ^2^Psychology Department, City College, The University of Sheffield International FacultyThessaloniki, Greece; ^3^Greek Association of Alzheimer's Disease and related DisordersThessaloniki, Greece; ^4^3rd Department of Neurology, Medical School, Aristotle University of ThessalonikiThessaloniki, Greece

**Keywords:** Alzheimer Disease, amnestic Mild Cognitive Impairment, electroencephalography, graph analysis, Relative Wavelet Entropy

## Abstract

Previous neuroscientific findings have linked Alzheimer's Disease (AD) with less efficient information processing and brain network disorganization. However, pathological alterations of the brain networks during the preclinical phase of amnestic Mild Cognitive Impairment (aMCI) remain largely unknown. The present study aimed at comparing patterns of the detection of functional disorganization in MCI relative to Mild Dementia (MD). Participants consisted of 23 cognitively healthy adults, 17 aMCI and 24 mild AD patients who underwent electroencephalographic (EEG) data acquisition during a resting-state condition. Synchronization analysis through the Orthogonal Discrete Wavelet Transform (ODWT), and directional brain network analysis were applied on the EEG data. This computational model was performed for networks that have the same number of edges (*N* = 500, 600, 700, 800 edges) across all participants and groups (fixed density values). All groups exhibited a small-world (SW) brain architecture. However, we found a significant reduction in the SW brain architecture in both aMCI and MD patients relative to the group of Healthy controls. This functional disorganization was also correlated with the participant's generic cognitive status. The deterioration of the network's organization was caused mainly by deficient local information processing as quantified by the mean cluster coefficient value. Functional hubs were identified through the normalized betweenness centrality metric. Analysis of the local characteristics showed relative hub preservation even with statistically significant reduced strength. Compensatory phenomena were also evident through the formation of additional hubs on left frontal and parietal regions. Our results indicate a declined functional network organization even during the prodromal phase. Degeneration is evident even in the preclinical phase and coexists with transient network reorganization due to compensation.

## Introduction

Alzheimer's Disease (AD) is regarded as a progressive, neurodegenerative disease with a relatively long pre-morbid asymptomatic period (Caselli et al., 2004). Although, no cognitive symptoms may be obvious this pre-morbid period is characterized by abnormal protein (amyloid-β/Aβ and hyperphosphorylated) production which results gradually in the formation of neurofibrillary tangles and neuritic plaques (Buerger et al., 2006). These alterations are particularly evident in brain areas crucial for the functional co-operation of distant brain regions (Delbeuck et al., 2003; Drzezga et al., 2011). Once clinical detection of AD is possible, based mostly on cognitive and daily functioning assessment, brain atrophy and thus functional impairments can be hardly inverted (Citron, 2010). It is therefore reasonable that research on Alzheimer's has focused on the reliable detection of early AD signs that precede functional and cognitive impairment (Sperling et al., 2011). As a result of this research effort, the term *Mild Cognitive Impairment* (MCI) (Petersen et al., 1999; Albert et al., 2011) was introduced to define a transition state between healthy aging and the clinical onset of dementia. It has been proposed that approximately 7% of people diagnosed with MCI eventually progress to Alzheimer's dementia (Mitchell and Shiri-Feshki, 2009). MCI is generally defined as memory impairment, despite normal daily functioning (Petersen, 2004). It has also been suggested that MCI is not a unitary disorder, but it can be further divided into various subtypes, indicating mostly the etiology: vascular, metabolic, amnestic, etc. (Petersen, 2004). Among them, the amnestic subtype it is considered to be a pre-clinical stage of AD (Dubois and Albert, 2004; Petersen, 2004; Vos et al., 2013).

The purpose of the present study is to investigate brain functional alterations that may characterize the amnesic subtype of MCI in order to aid to the early diagnosis of AD. Functional analysis can be performed by employing neurophysiological features derived from electroencephalographic (EEG) rhythmic activity (Moretti et al., 2011, 2012, 2013). During the last decade, a new category of metrics, based on the topological architecture of brain connectivity, has been introduced to estimate the organization characteristics of brain networks (Bassett and Bullmore, 2006). Connectomics may provide valuable information regarding the quantification of the network properties (Van Dijk et al., 2010). They employ either structural or functional connectivity to construct brain networks. Network properties are then computed through graph theory analysis (Stam and Reijneveld, 2007). A major notion of graph theory is that of small-world, which describes how efficient and cost-effective the network is. Computation of the small-world value considers both the quality of local information processing and the co-operation of distant brain regions. Therefore, brain networks with large small-world values are densely locally clustered, and at the same time employ the optimal number of distant connections to process information more efficiently and with lower information cost (Bassett and Bullmore, 2006; Bullmore and Sporns, 2009).

During the last few years, several research efforts have provided evidence of loss of “small-worldness” and reorganization of the brain networks due to neurodegeneration (Stam et al., 2009; Sanz-Arigita et al., 2010; Zhao et al., 2012). The majority of these studies have compared healthy adult participants with dementia patients. To the best of our knowledge, only a couple of studies so far have analyzed small-world networks in MCI patients using magnetoencephalography (MEG) (Buldú et al., 2011) and functional MRI (Seo et al., 2013), respectively. Both studies found abnormally increased and decreased synchronization in (pre)frontal and parieto-occipital regions respectively in the MCI patients compared to the healthy adults. More specifically, MCI patients showed an abnormal synchronization increase in comparison to healthy controls during the execution of memory tasks. It was associated with high energy expenditure which may be attributed to the existence of compensatory mechanisms recruited by MCI patients toward the successful execution of cognitive functioning (Buldú et al., 2011). Another study reported loss of functional integration as quantified by the characteristic path length (Seo et al., 2013). However, findings are quite contradictory among studies, since some of them report either no significant changes (Seo et al., 2013) or increased characteristic path lengths for the patients suffering from Alzheimer's (Yao et al., 2010; Zhao et al., 2012). Seo et al. reported diminished information transfer among brain regions for both MCI and MD participants due to functional impairment of the hubs, which are network nodes connecting local networks and facilitating global information processing (Seo et al., 2013).

Binary brain networks are usually constructed by applying a threshold to the metric quantifying the synchronization between two network nodes. A pair of nodes is connected with a network edge when the synchronization degree between these two nodes exceeds the pre-defined threshold. The ratio of the number of network connections (edges) to the number of possible edges is defined as the network's density. The threshold selection is important for the network formation. Application of a fixed threshold value is vulnerable to inter-participant variability, thereby resulting in networks with different density values. The latter influences the network properties (characteristic path length, mean cluster coefficient) and computation of small-worldness cannot be easily performed. Aiming to face this methodological limitation, recent studies adopted the adaptive threshold selection for each participant in order to produce brain networks of fixed density. So, all graphs may have the same number of edges; in this way, group comparison is facilitated. However, there is not yet a gold standard for selecting a fixed density-based threshold. Therefore, the full network analysis is repeated over a density range. Adopting the aforementioned methodological approach, a more recent study recruited a large number of participants (94 controls, 183 MCI patients and 216 MD patients) employing fluorodeoxyglucose positron emission tomography (FDG-PET) (Seo et al., 2013). In addition to the global network analysis through the small-world property, this research investigated the vulnerability of the network hubs. The results showed that both MCI and AD groups had lower local clustering compared to healthy controls. Both pathological groups demonstrated vulnerability of the nodes that are crucial for the information transfer within the brain network (functional hubs). These hubs were mainly associated with the Default Mode Network (DMN).

It has been, therefore, suggested that brain networks are altered in people with neurodegenerative brain disorders, and this alteration is usually evidenced as diminished local processing and disrupted co-operative activity among distant brain regions. However, most focus has so far been placed on the clinical AD phase, while research on functional network analysis during the preclinical (aMCI) phase is scarce. Electroencephalographic (EEG) analysis may provide a direct window of brain functioning. Its excellent temporal resolution could offer a reliable way of quantifying brain co-operative activity during the resting-state condition. Aiming to enhance the understanding of the disease progression and to propose contemporary mathematical tools able to identify early functional disorganization phenomena, this piece of work employs EEG recordings and attempts to answer the following research questions:

1. Is there any evidence of functional disorganization in aMCI, which can be differentiated from healthy aging?
2. Is there a relationship between network architecture and general cognitive state?
3. Are there any significant differences in the network disorganization among aMCI and MD groups? If yes could we also detect any recruitment of additional brain regions during the prodromal phase?

According to previous evidence we expect that aMCI patients will exhibit significant network deficiency as compared to healthy older adults (Buldú et al., 2011; Seo et al., 2013). We also expect network disorganization in aMCI to result mostly from a reduced local information processing capacity as expressed by *the mean cluster coefficient* value (Seo et al., 2013). Since previous research has shown that the *characteristic path length*, which quantifies information integration and transmission, remains relatively stable across different neurodegenerative phases in AD (Seo et al., 2013), we do not expect this parameter to be affected in the groups of aMCI or MD, relative to the group of healthy controls. The interplay among reduced local processing and relatively stable information transmission is hypothesized to affect the global network functional organization. Since aMCI is regarded as the earliest AD phase, we expect that aMCI individuals would exhibit network deficiencies similar to those of patients suffering from AD and these alterations would be mainly manifested as a disrupted small-world property and abnormal local information processing (Buldú et al., 2011; Seo et al., 2013). It is also expected that the global network architecture would be correlated with neuropsychological tests estimating the generic cognitive status. Finally, the two pathological groups, aMCI and MD, would probably show a vulnerability of network nodes that are crucial for information transfer and cognitive functioning (Seo et al., 2013). These nodes are defined as network hubs and their robustness is estimated by centrality metrics. However, since the aMCI patients relatively preserve their cognitive and daily functioning, compensatory mechanisms may invoke a network reformation in that stage. Therefore, we hypothesize that functional hubs occurred in the healthy brain become less robust and additional hubs are formed during the aMCI phase (Qi et al., 2010).

## Materials and methods

### Participants

Twenty-three cognitively healthy older adults, 17 aMCI and 24 mild demented (MD) individuals participated in the present study. All of them went through a neuropsychological assessment which was part of the screening process for the *Long Lasting Memories* (LLM) project. LLM was a multi-centric, European Commission-funded project that proposed a computerized intervention of cognitive and physical exercise in order to promote independent living of senior participants (www.longlastingmemories.eu) (Bamidis et al., 2011; González-Palau et al., 2014). Screening took place 1–14 days before the participants' enrollment to the training (Frantzidis et al., 2014). Prior to neurophysiological acquisition, all participants were informed about the study and signed an informed consent form. The study was approved by the ethics committee of the Greek Association of Alzheimer's Disease and Related Disorders.

The following Table (Table 1) reports information about the participants' age and generic cognitive status as estimated by the Mini Mental State Examination (MMSE) and the Montreal Cognitive Assessment (MoCA) test (mean values ± standard deviation), and the number of participants per group. The groups were matched on age and male-to female ratios (all *p*_*s*_ > 0.05).

**Table 1:** **Mean age, sex and cognitive status for the participants of each group enrolled in the present study**.

**Group**	**Age**	**Number of participants**	**MMSE**	**MoCA**
*Healthy*	68.0 ± 5.5	23 (6 males)	28.0 ± 2.1	26.0 ± 2.4
*aMCI*	68.6 ± 2.7	17 (4 males)	25.6 ± 2.2	25.6 ± 2.2
*MD*	72.3 ± 6.3	24 (7 males)	22.3 ± 2.5	17.3 ± 4.3

### Neuropsychological examination

The neuropsychological examination included a complete set of tests aiming to assess the participant's generic cognitive status as well as other specific cognitive domains (verbal memory, executive functions, independent living, etc.) that are essential to the diagnostic procedure and the group formation. A detailed list may be found in Bamidis et al. (2012).

### Medical examination

Medical examination consisted of a full blood count, biochemical tests and examination of various parameters such as thyroid hormones, anti-thyroid auto-antibodies, homocysteine and folic acid levels. The Erythrocyte Sedimentation Rate (ESR) was also estimated. Neuroimaging examination either through MRI or Computerized Tomography (CT) was adopted to exclude participants suffering from various parameters that may influence the study results (e.g., cancer of the central nervous system, hypercholesterolemia, etc.). Finally, the participants visited a doctor involved in the current study. Their medical and family history as well as their current and past medication were recorded.

### Diagnostic procedure

A dementia expert neurologist performed the diagnosis of each participant considering the aforementioned examinations. AD diagnosis was performed according to both the DSM-IV and the criteria of the National Institute of Neurological and Communicative Disorders and Alzheimer's Disease and Related Disorders (NINCDS-ADRDA) (McKhann et al., 1984). Patients suffering from aMCI, met Petersen's criteria (Petersen, 2004). The study groups were matched according to the baseline demographic variables (age and sex). This study was focused on MCI patients suffering from multiple domains and having as major problem that of memory impairment (Petersen, 2004). This group of patients would be referred as aMCI in the remaining of the manuscript, but merely for briefness, as the most appropriate term is that of multiple domain + amnestic MCI (Petersen, 2004).

### EEG analysis

#### Data acquisition and pre-processing

Neurophysiological data acquisition was performed through a Nihon Kohden JE-207A. The device was equipped with 57 active electrodes attached on a cap fitted to the scalp (EASYCAP). There were also 2 reference electrodes attached to the mastoids and a ground electrode placed at a left anterior position. Both vertical and horizontal electrooculograms (EOG) and electrocardiographic (ECG) activity were recorded through bipolar electrodes. Electrode impendances of brain signals, ground electrode and references were kept lower than 2 KΩs. The sampling rate was set at 500 Hz. Participants were sitting in a comfortable armed chair located in a quiet room with minimal ambient light. They were instructed to remain calm, with their eyes closed, for 5 min at least.

The brain electrodes were re-referenced using the two reference electrodes located on the mastoids in a way described also in Frantzidis et al. (2014). Then, Butterworth digital filtering of 3rd order was performed through a high pass filter with cut-off frequency at 1 Hz and a notch filter centered on 50 Hz. Independent Component Analysis (ICA) was then employed to remove artifactual components. Finally, visual inspection was performed to eliminate data segments contaminated with noise. The aforementioned pre-processing procedure was performed through the Matlab Signal Processing Toolbox and the EEGLAB graphic user interface (Delorme and Makeig, 2004).

#### Synchronization analysis

**Figure 1:**
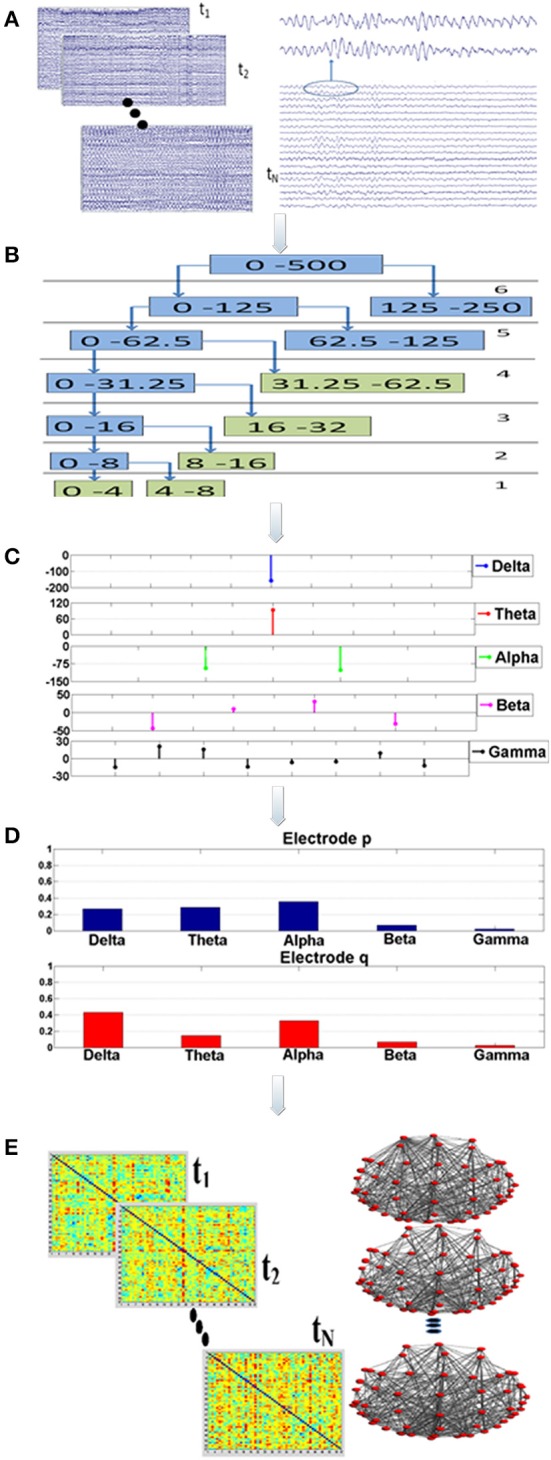
**Visualization of the proposed analysis framework: there are five main analysis steps (A–E)**. Firstly, a randomization, bootstrap technique^*^ is employed during step “A” to select (*N* = 75) multiple, artifact free data epochs. This bootstrap technique is implemented through a generator of random numbers which produces choices of data epochs. Each epoch lasts for 20 s. For each data segment and for each electrode the Orthogonal Discrete Wavelet Transform (ODWT) is applied through an iterative, recursive decomposition scheme (Step “B”). The ODWT framework results in the estimation of the wavelet coefficients for each frequency band and for each epoch. The computations are performed on 128 ms intervals, resulting in one (1) wavelet coefficient for the slow (delta, theta rhythms), two (2) coefficients for the alpha, four (4) for beta and eight (8) for gamma (Step “C”). These coefficients are then squared in order to express the rhythm's energy. So, the relative energy contribution of each frequency band is then computed by dividing the energy of each rhythm by the total EEG energy during Step “D.” The Relative Wavelet Entropy (RWE) is then computed for each electrode pair. The RWE provides a directed metric of the co-operative degree among two electrodes. Then, synchronization matrices based on the RWE values are formed. These matrices are then thresholded and directed, non-weighted networks are formed. These networks are employed toward the estimation of both global (small-world, characteristic path length, mean cluster coefficient) and local (relative betweenness centrality) characteristics.

The synchronization analysis involved 75 epochs of artifact-free, continuous data of high quality (Figure 1; Step “A”). The duration of each epoch was set at 20 s, since it was demonstrated in a previous work that this time interval is sufficient for extracting the synchronization degree in a robust way (Gudmundsson et al., 2007; Hsu et al., 2012; Frantzidis et al., 2014). Aiming to avoid methodological and sampling errors, the epoch selection was performed in a completely randomized way. More specifically, a random number generator output choices of continuous, artifact-free epochs to be used for the unbiased synchronization analysis.

Synchronization analysis (Figure 1; Steps “B-D”) aimed firstly at the robust extraction of activity for each frequency band for every electrode (Step “B”), its relative energy contribution (Step “C”) and finally at the quantification of the co-operative degree among pairs of electrodes (Step “D”) by employing wavelet analysis through the Orthogonal Discrete Wavelet Transform (ODWT). Wavelets were subjected to scaling and translation in order to extract both frequency and time-dependent components with optimal resolution. ODWT also involved an iterative decomposition scheme through recursive low-pass filtering for computing the wavelet coefficients of the five frequency bands in a way that discarded redundant information, while allowing the perfect reconstruction of the whole EEG. Wavelet coefficient amplitudes indicated the degree of correlation among the wavelet and the signal, while the sign of each coefficient represented the type of correlation (positive/negative). All computations were implemented through Matlab functions (Wavelet Toolbox).

The family of 5th order bi-orthogonal wavelets was selected as the mother wavelet (Frantzidis et al., 2010, 2014). This specific type of wavelets was selected due to its resemblance with common EEG waveforms and its attractive mathematical properties (e.g., semi-orthogonality, symmetry, smoothness and maximum time-frequency resolution). Therefore, phase distortion and discontinuity effects are avoided (Unser et al., 1992; Quian Quiroga and Schürmann, 1999; Frantzidis et al., 2010). Each epoch was divided in non-overlapping windows of 128 ms duration and computations were performed for each window, in which the first step was the computation of the wavelet coefficients using a decomposition scheme of *j* = 1 … 5 levels. Multiple coefficients (*k* = 1 … K) were calculated for each decomposition level, except of the last one (*j* = 5). The energy of each frequency band (*E*_j_) was estimated by firstly squaring and then summing the wavelet coefficients (*C*_*k*_) corresponding to each rhythm:

(1) $$E_{j}=\sum_{k\;=\;1}^{K}\left|C_{k}^{2}\right|,\text{j}=1\ldots 5$$

A simple summation of all energies for each frequency band provided the total EEG energy:

(2) $$E_{tot}=\sum_{j\;=\;1}^{5}E_{j}$$

Relative energies at each frequency band were estimated by dividing each absolute energy value *E*_j_ with the total energy *E*_tot_.

These computations involved the 57 brain electrodes that formed 3192 electrode pairs. The number of electrode pairs was computed as follows: each one of the 57 electrodes was compared with all the other electrodes. Since the metric is a directional one the electrode pair (p, q) is different from the pair (q, p). Therefore, we had 57 × 57 = 3249 comparisons. Among these there are 57 pairs that compare the same electrode (p, p). That electrode pairs are not meaningful and were subtracted. Therefore, the total number of electrode pairs is 3249−57 = 3192. The mathematical framework resulted in a probabilistic energy distribution (Figure 1; Step “D”) for each one of the 57 electrodes participating in the 3192 electrode pairs. The probabilistic energy distribution of each electrode was consisting of contributions of each frequency band (a positive number) to the total energy of a specific electrode for a given time period (window duration). Since, these numbers quantify the energy ratio of each frequency band to the total EEG energy, their summation was equal to one (Rosso et al., 2001; Frantzidis et al., 2010, 2014). Finally, the synchronization degree among each electrode pair was computed through the notion of the Relative Wavelet Entropy (RWE) which represented the co-operation degree of the generalized rhythmic activity among two distinct electrode sites (Figure 1; Step “E”). Since there were *N* = 57 electrodes, the dimension of the synchronization matrix is *N* × *N* = 57 × 57. In case of two electrodes with energy distributions p_j_ and q_j_, the synchronization degree (RWE value) was given by the following formula (the smaller the RWE value, the greater the synchronization):

(3) $$RWE=\sum_{j\;=\;1}^{5}p_{j}\times \ln\left(\frac{p_{j}}{q_{j}}\right)$$

Therefore, the main diagonal of the synchronization matrix contained zero values (comparison of a signal with itself). As mentioned earlier, these 57 electrode pairs do not participate in the computations.

### Network analysis

#### Synchronization matrix thresholding

Synchronization matrices were then passed through a threshold to be transformed into binary, directed brain graphs (Figure 1; Step “E”). Aiming to avoid the influence of methodological limitations posed by brain networks of varying density, the selection of an adaptive threshold was preferred. This choice ensured that the brain network of each participant would have the same number of edges. So, both global and local network properties (small-word value, characteristic path length, mean cluster coefficient, global efficiency and normalized relative betweenness) were quantified for each participant and for four (4) fixed density ranges (500, 600, 700, 800 edges). The number of edges corresponded to 15.39, 18.47, 21.55, and 24.62% density values, respectively. Analysis, over a wide density range was preferred since there is lack of a golden standard for density selection. It was also unknown whether the influence of neurodegeneration phenomena could be detected in both low and high density networks. The following section provides a brief description of these network characteristics.

#### Description of network parameters

A network is represented by a graph that consists of nodes and edges. Each electrode represents a node, which is connected with another one through an edge (Bassett and Bullmore, 2006). These edges may be directed (directed graphs) or not (undirected graphs). The size of a graph depends on the total number of nodes, while its degree is the mean value of edges per node. The distance between two nodes is computed by the total number of edges of the shortest path needed to reach from one node to another. The *characteristic path length* (L) is computed by the mean (or in some cases median) value of the shortest paths among all pairs of nodes (Bassett and Bullmore, 2006; Stam and Reijneveld, 2007; Bullmore and Sporns, 2009; Stam et al., 2009).

To calculate the *cluster coefficient, C*, for each node, a 3-step procedure is followed:

- Immediate neighbors of (those directly connected with) a given node are identified.
- The number of connections among immediate neighbors is computed (existing connections).
- C, for a given vertex/node, is then computed as the ratio of the number of existing connections to the total number of all possible connections in the immediate vertex neighborhood, ranging from zero to one. Finally, the mean cluster coefficient is computed as the mean value of all cluster coefficient values (Lithari et al., 2012).

The small-world property, introduced by Watts and Strogatz (1998), is usually employed to characterize network architectures by means of dense clustering of local connections, as well as, short characteristic path lengths achieved by a few long-range connections, thereby facilitating the fast and efficient information transfer among all network nodes. Thus, small-network topologies offer an attractive model for brain network connectivity quantification, since they combine strong local information processing (high cluster coefficient value) with fast and efficient, global information transfer through small characteristic path length (Sanz-Arigita et al., 2010; Buldú et al., 2011; De Haan et al., 2012; Lithari et al., 2012; Seo et al., 2013); estimating the small-world property involves computation of the network characteristic path length (L) and mean cluster coefficient (C), as well as, the comparison with the corresponding properties (*L*_*rand*_ and *C*_*rand*_) of a random graph containing the same number of nodes (N), edges (K) and degree of distribution as shown by formulae (4) and (5):

(4) $$L_{rand}\;=\frac{\ln(N)}{\ln\left(\frac{k}{N}-1\right)}$$

(5) $$C_{rand}\;=\frac{\left(\frac{k}{N}\right)}{N}$$

Then the ratios λ = *L*/*L*_*rand*_ and γ = *C*/*C*_*rand*_ are combined to retrieve the small world property (sigma), sigma = γ/λ. Small-world networks exhibit sigma values greater than one (Bassett and Bullmore, 2006).

The local nodal metric of betweenness centrality, B_i_ (for each node *i* = 1 … *N*), is also employed to investigate whether the age-related neuro-degeneration affects nodes with a functionally significant role (hubs) or not. B_i_ is defined as the number of shortest paths from all nodes to all others that run through node i. Therefore, it quantifies the amount of information transferred through node i. To normalize raw B_i_ values, the value for each node is divided by the mean B_i_ value of the whole network. In this way, and when B_i_ is greater than 1.5, a node can be regarded as a functional hub. This parameter setting was adopted from a previous study (Seo et al., 2013). The threshold value, which was a strict one, was the same.

## Results

### Global characteristics and their alterations in aMCI and MD

**Figure 2:**
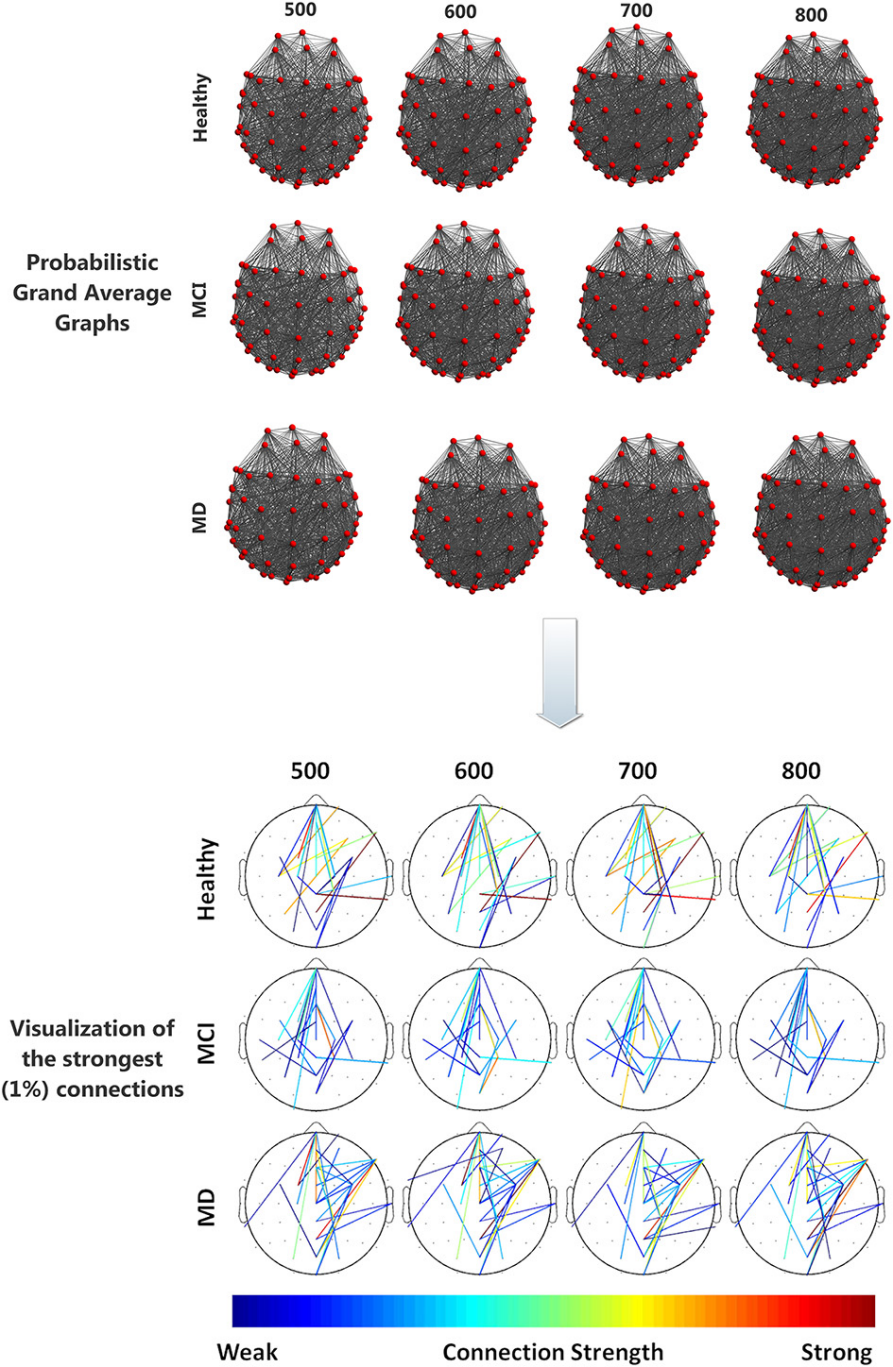
**(Top)** Visualization of grand average brain graphs for each one of the three study groups (Healthy, MCI, MD) and for each edge density value (500, 600, 700, 800). **(Bottom)** visualization of the strongest (1%) network connections and their (edge) strengths, depicted through a colorbar.

All groups (Healthy, aMCI, MD) demonstrated small-world characteristics (σ > 1) over the entire range of densities. Tables S1–S3 in Supplementary Material show means and standard deviation as a function of group and density for each network characteristic. Figure 2 illustrates the mean (grand average) brain networks for each one of the three groups (Healthy, aMCI, MD) as well as the networks with the strongest (1%) connections only. The visualization is performed for the entire density range employed in the study (*N* = 500, 600, 700, 800).

**Figure 3:**
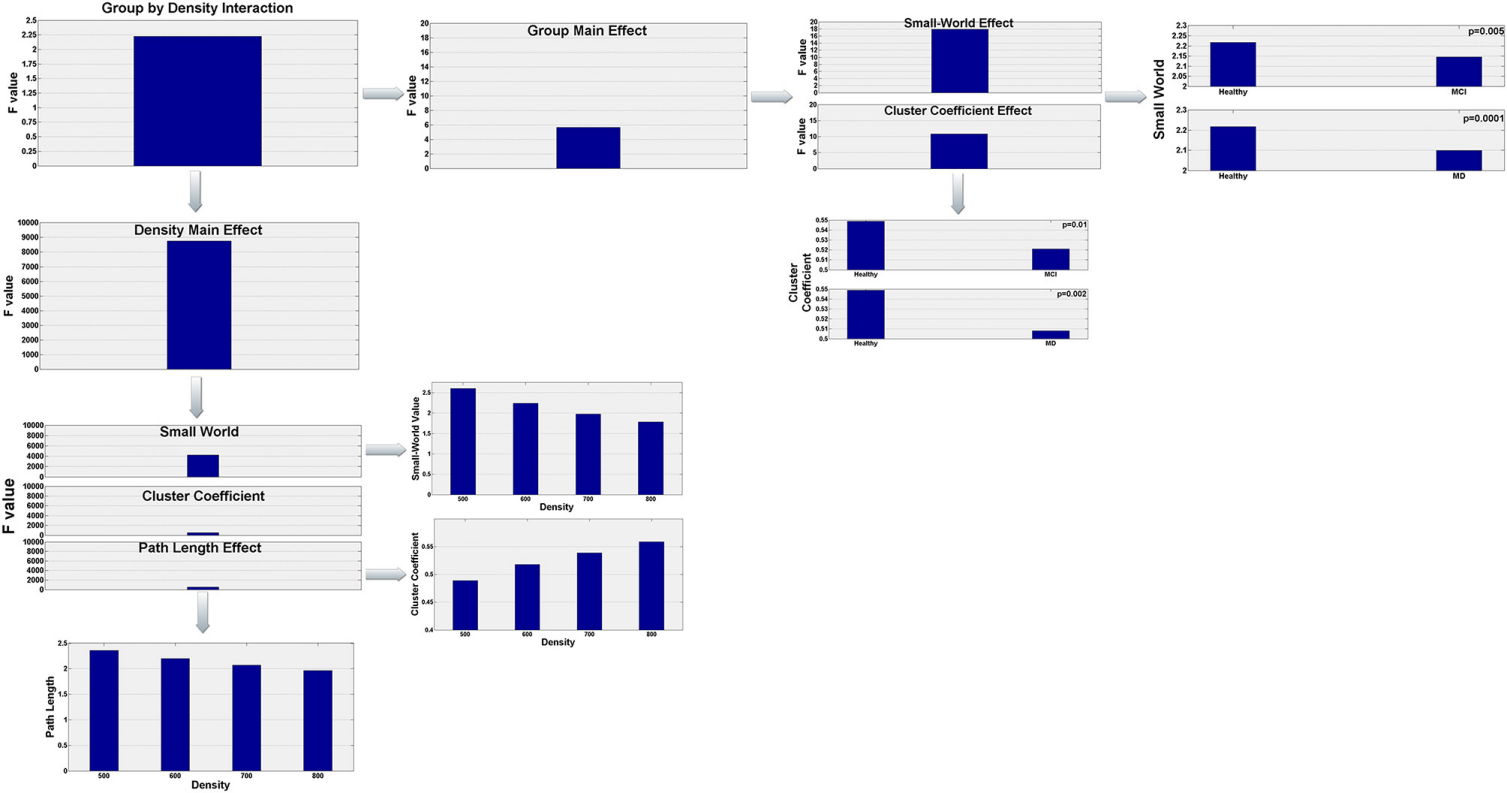
**Visualization of the statistically significant network parameters results (Small-World, Characteristic Path Length, Cluster Coefficient)**. Results refer to network differences among the three groups (Healthy, aMCI, MD) and are dependent on the density parameter (*N* = 500, 600, 700, 800). More specifically, statistical analysis demonstrated a significant group by density interaction. Both group and density main effects were further analyzed in order to highlight how global network characteristics differ among the three groups and how these parameters are affected by the density of the graph.

To analyze the data we conducted a 3 × 4 by (3) MANOVA with group (Healthy, aMCI, and MD) as the between subject factor, density (500, 600, 700, 800) as the within subject factor, and the 3 interrelated dependent variables (small-world value, C and L). Using Pillai' trace, there were significant effects of group [*V* = 0.44, *F*_(6, 120)_ = 5.63, *p* < 0.0001], and density [*V* = 0.999, *F*_(9, 53)_ = 8766.855, *p* < 0.0001], and a significant group by density interaction [*V* = 0.541, *F*_(18, 108)_ = 2.225, *p* = 0.006] on small world property, Cluster Coefficient and Length path. Separate 2 × 4 ANOVAs on the 3 outcomes variables revealed a significant^1^ main effect of group for the small world property, *F*_(2, 61)_ = 17.92; *p* < 0.0001, and the Cluster Coefficient *F*_(2, 61)_ = 10.83; *p* < 0.0001. Also there was a main effect of density for the three outcomes variables, small world property, *F*_(3, 183)_ = 4236.71, *p* < 0.0001; cluster coefficient, *F*_(3, 183)_ = 524.28; *p* < 0.0001, and path length, *F*_(3, 183)_ = 529.10; *p* < 0.0001. Tukey HSD *post-hoc* comparisons for the group factor showed significant differences between the Healthy controls group (2.217) and the aMCI (2.146) and MD groups (2.099) for the Small World property, *p* = 0.005 and *p* = 0.0001, respectively. There were no significant differences between the aMCI and the MD groups. Similarly, for the Cluster Coefficient, there were significant differences between the Healthy controls group (0.549) and the aMCI (0.521), and MD groups (0.508), *p* = 0.01 and *p* = 0.0002, respectively. Again, there were no significant differences between the aMCI and the MD groups. For the Small World property, Tukey HSD *post-hoc* comparisons showed significant differences between the four density values, 500 (2.609), 600 (2.245), 700 (1.977), and 800 (1.784), all *p*s < 0.0001. That is, the Small World property value decreased as the density value increased. The same pattern was observed for the Path length (Mean_500_ = 2.357, Mean_600_ = 2.199187, Mean_700_ = 2.071634, Mean_800_ = 1.962972). The Path Length value decreased as the density value increased, all *p*s < 0.0001. Finally, for the Cluster Coefficient, Tukey HSD *post-hoc* comparisons showed again significant different between all density conditions (Mean_500_ = 0.489, Mean_600_ = 0.518, Mean_700_ = 0.539, Mean_800_ = 0.559), all *p*s < 0.0001. However, the Cluster Coefficient value increased as the density increased. The statistically significant results regarding the graph parameter differences for the three groups are visualized in Figure 3.

**Figure 4:**
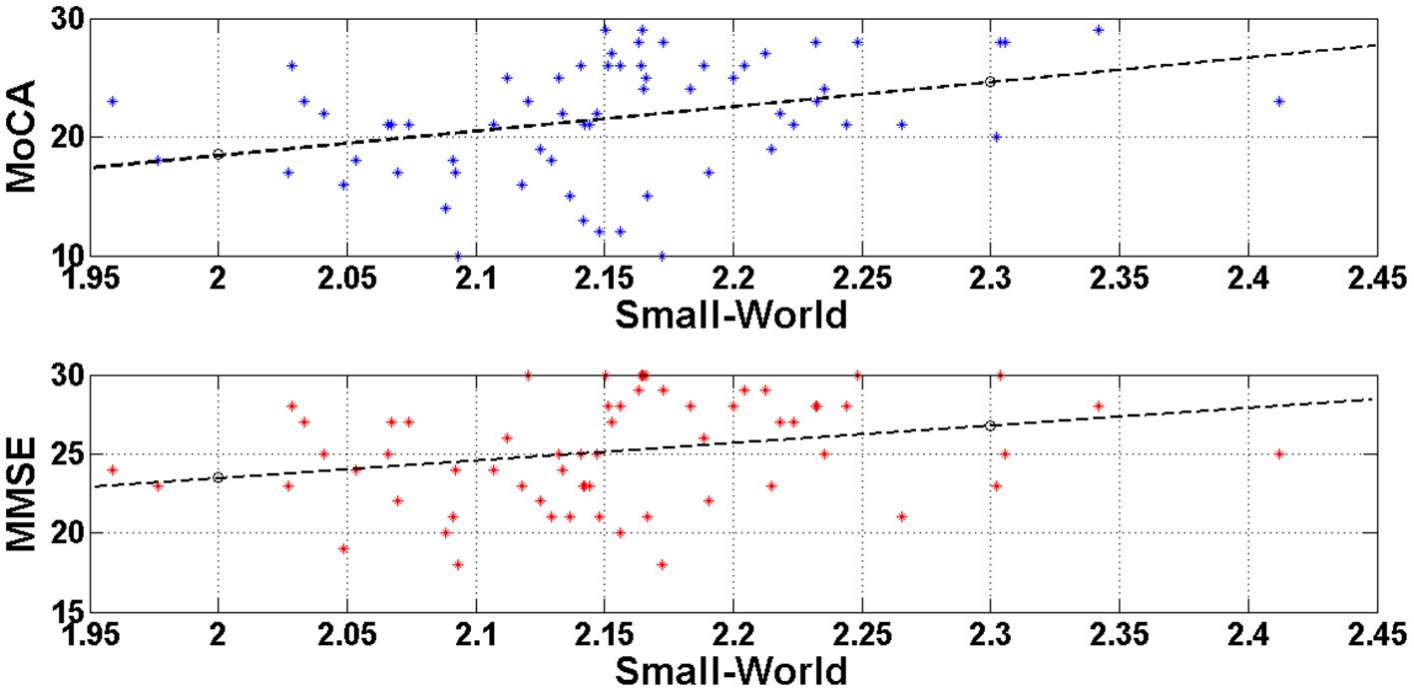
**Visualization of the linear correlation among the network architecture (Small-World) with the generic neuropsychological tests**. The correlation degree was estimated through the Pearson coefficient and was greater for the MoCA (*r* = 0.470) and lower for the MMSE (*r* = 0.367). Both correlations would be characterized of medium strength and may indicate that deficient generic cognition may be attributed to disturbances of the resting-state brain networks.

In order to test if there was a linear relationship between cognitive status as measured with the MMSE and the MOCA, and the small-world property value we computed Pearson's correlations. The analyses showed a significant positive correlation between the MMSE scores and the Small World property, *r* = 0.367, *df* = 63, *p* = 0.003, and between the MoCA scores and the Small World property, *r* = 0.470, *df* = 63, *p* < 0.0001. Figure 4 illustrates these correlations through scatter plots of the MoCA/MMSE data distributions against the Small-World data. More specifically, the horizontal axis (Small-World value) was estimated as the mean of the four small-world values of each edge density range (*N* = 500, 600, 700, 800). Since this metric quantifies the linear correlation among two variables, the statistically significant results indicate a linear correlation of medium strength among the network architecture and the performance on the generic neuropsychological estimation. This finding may demonstrate that the degree of network performance may reflect deficiency in generic cognitive processing.

### Functional hub identification

**Figure 5:**
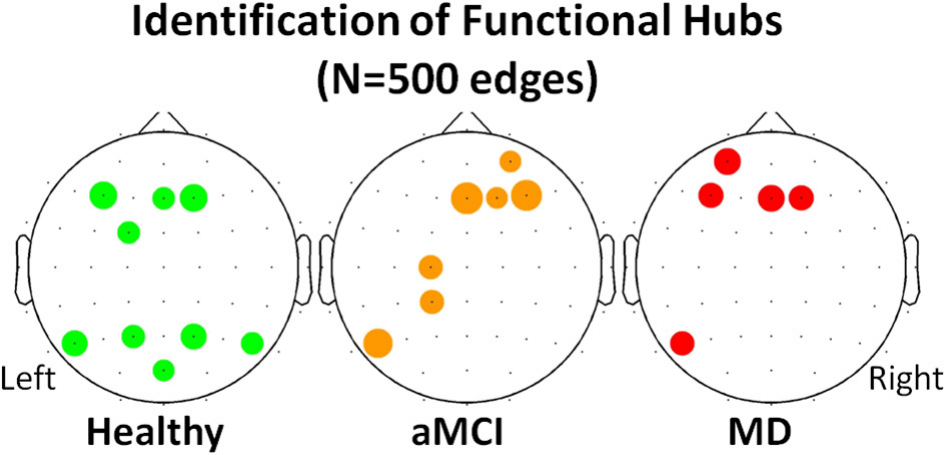
**Visualization of the functional hubs identified for each one of the three groups (Healthy, aMCI and MD)**. The hub identification was based on the normalized relative betweenness centrality value. According to a previous study, a node is defined as a hub when its centrality value is greater or equal to 1.5 (Seo et al., 2013). This threshold is a strict one. Therefore, the analysis was performed on the lower density range (*N* = 500). A small density value is more likely to result in a greater number of functional hubs. The hubs (names and locations) are visualized in a sensor level for each group. The hub strength is also reported through its relative betweenness centrality (b_i_) value.

Following the work of Seo et al. (2013), electrodes were identified as functional hubs based on their standardized Bi value (*Bi* ≥ 1.5). The identification procedure was performed for each one of the three groups (Healthy, aMCI, MD) for the density condition *N* = 500 edges. Visualization of the functional hubs is presented in Figure 5.

The mean normalized B_i_ value of the identified hubs of the healthy group computed for all participants and all groups (Table 2). These values were then submitted to One-Way ANOVA with group as the between subject factor. Results showed a significant main effect of group, *F*_(2, 61)_ = 5.87; *p* = 0.005. Tukey HSD *post-hoc* comparisons showed significant differences between the Healthy Controls group (Mean = 1.849; *SD* = 0.463) and the aMCI (Mean = 1.365; *SD* = 0.523) and MD groups (Mean = 1.503; *SD* = 0.435), *p* = 0.006, and *p* = 0.037, respectively. There were no significant differences between the aMCI and the MD groups. That is, both aMCI and MD groups had significantly lower nodal strength of functional hubs as compared to healthy controls.

**Table 2:**
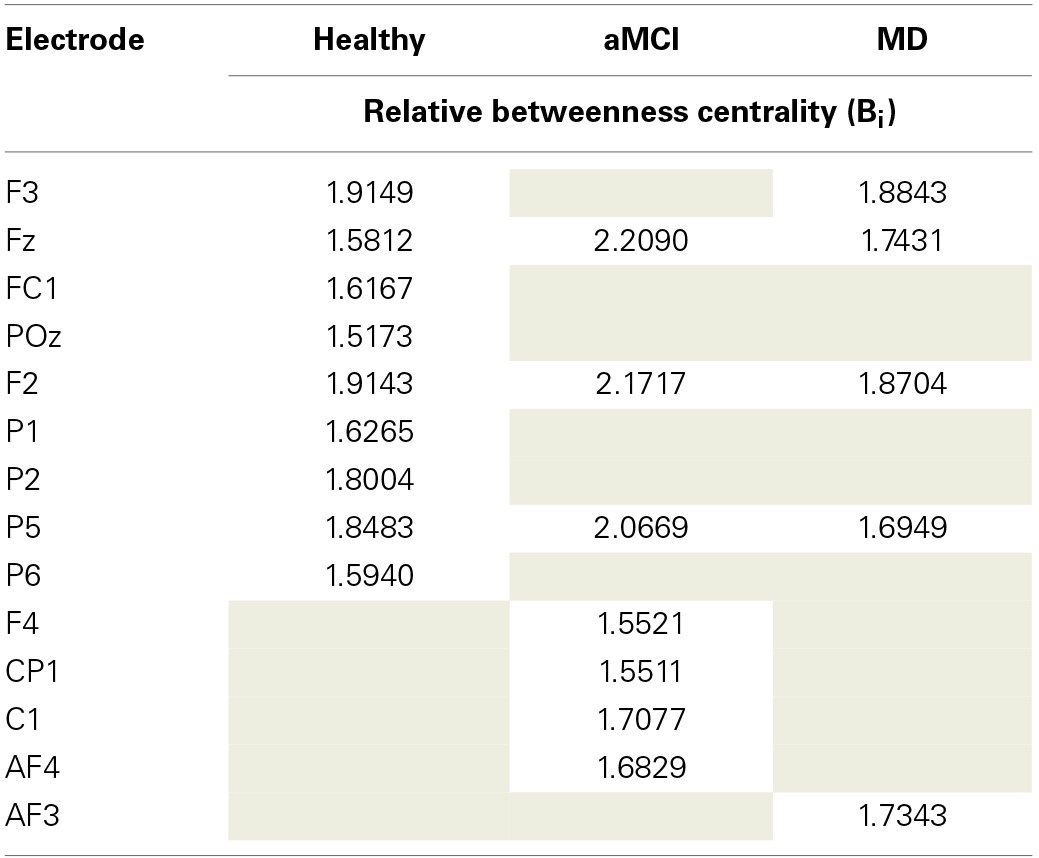
**Description of the functional hubs identified in the three (Healthy controls, aMCI, MD) groups**.

*The functional hubs were identified in terms of their normalized relative betweenness centrality value (B_i_). Nine (9) hubs covering mainly frontal and parietal areas were identified in the healthy controls. Seven (7) hubs located mainly on right frontal and left posterior areas were identified in the aMCI patients. Five (5) hubs located mainly on frontal and on left parietal areas were identified in the MD group*.

To investigate whether neurodegeneration induced the additional recruitment of anterior, bilateral regions we proposed the computation of the Anterior Hub Ratio (AHR) as the ratio of the nodal significance of *left anterior/right anterior* functional hubs in terms of relative betweenness centrality. This AHR metric quantifies the functional interplay among anterior hemispheres. It was based on the hub identification described previously. So, the nominator included the left anterior hubs (F3, FC1, AF3, F1, FC3) and the denominator included the right anterior hubs (F2, F4, Fz, Afz, FCz, FC2, FC4). One-Way ANOVA with group as the between subject factor showed a significant main effect of group [*F*_(2, 61)_ = 3.27, *p* = 0.045]. Tukey HSD *post-hoc* comparisons showed significant differences only between the group of Healthy controls (Mean = 0.517; *SD* = 0.327) and the group of aMCI (Mean = 1.179, *SD* = 1.279), *p* = 0.0430. There were no significant differences between the MD group (Mean = 0.943, *SD* = 0.868) and the other groups.

## Discussion

To investigate functional network organization in aMCI and MD, we employed graph analysis of resting-state electroencephalographic data. The results of the global characteristics indicated that all three (3) groups demonstrated small-world characteristics. However, both aMCI and MD patients showed reduced global network properties (small-world value and mean cluster coefficient) in comparison with the healthy controls. This result is in agreement with previous studies that showed a loss of optimal network organization in AD patients (Stam et al., 2007; He et al., 2008; Supekar et al., 2008; Zhao et al., 2012) and the general category of MCI (Yao et al., 2010; Seo et al., 2013).

Previous studies with MCI participants yielded contradictory results. Specifically, Yao et al. performed cortical network analysis through gray matter volume characteristics obtained from MRI (Yao et al., 2010). The study design included 98 healthy controls, 113 MCI participants and 91 AD patients. The MCI group exhibited intermediate small-world values. Further analysis, revealed that comparison of the MCI network characteristics (cluster coefficient and characteristic path length) either with the healthy or the AD group did not reach statistical significance. Nevertheless, in a more recent study recruiting 94 healthy controls, 183 MCI and 216 AD patients, graph analysis was performed through FDG-PET data. It was found that both MCI and AD patients demonstrated lower cluster coefficient than healthy controls, while the characteristic path length was not affected. The study also reported that MCI participants exhibited the lower cluster coefficient values. (Seo et al., 2013). Aiming to avoid the heterogeneity of the entire MCI spectrum, we tested only patients suffering from the amnestic subtype which is considered to be a pre-stage of AD (Dubois and Albert, 2004; Petersen, 2004). Our results support that there are no differences between aMCI and MD patients in terms of network function (small-world, mean cluster coefficient and characteristic path length). That is, both groups of patients showed the same pattern of network property breakdown as compared to Healthy controls. Since these two groups are diagnostically different, we consider these results in terms of compensatory mechanisms. That is, we propose that compensatory mechanisms are preserved in aMCI, and that loss of these mechanisms may lead to progression to mild dementia. This hypothesis would be in agreement with our finding of additional hub formations in the group of aMCI.

However, absence of statistically significant findings regarding the characteristic path length seems to be in contradiction with the only other (prior) study that has investigated network organization in a group of 37 aMCI patients (Wang et al., 2013). That study employed fMRI recordings combined with frequency-dependent wavelet based correlation analysis and reported abnormally increased *path length characteristic* in the group of aMCI. This contradiction may be attributed to the much smaller number of participants that our study enrolled in both groups. Another possible explanation may be that Wang et al. extracted frequency-dependent brain networks, while our methodology received the entire EEG range as input and computed the co-operative degree in terms of frequency-based similarity of the probability distribution among electrode pairs.

In addition, we found a statistically significant positive correlations between small-worldness and cognitive status as measured with MMSE and MoCA. That is, the more cognitively deteriorated (lower scores in MMSE and MoCA) the patients are, the less optimal the network organization is (lower small world values). This finding is also in agreement with a previous finding of a positive correlation between characteristic path length values and MMSE scores in a group of AD patients (De Haan et al., 2009). We deem our findings to be important in this sense, as we extended those results to small-world property which better quantifies the global network performance. In addition we included a larger sample with healthy adults, aMCI and MD individuals. Overall, these findings suggest network analysis may be used as a tool for detecting age-related pathological disorders due to neurodegeneration.

Local network analysis was performed through the identification of those nodes that were important for the network organization. Those nodes were named functional hubs. The hub definition was based on the amount of information flow the nodes transfer. The normalized betweenness centrality was previously proposed to be a robust metric of the hub strength (Seo et al., 2013). The results demonstrated that healthy hubs seem to be preserved to some extent during the aMCI and mild dementia phase. However, they are functionally impaired, as it is demonstrated by statistically significant decreases in terms of betweenness centrality. This finding may be indicative of deficiency due to neurodegeneration and impaired functional connection of distant brain regions. Apart from hub strength reductions, aMCI participants formed additional hubs especially in the left frontal and parietal regions. The hub formation may be attributed to compensatory mechanisms (Cabeza et al., 2002; Hämäläinen et al., 2007; Qi et al., 2010); according to these studies healthy elderly recruit additional frontal and parietal regions during memory processes (Cabeza et al., 2002), while increased frontal activation of MCI patients compared to controls is observed through fMRI recordings even in the resting state condition (Hämäläinen et al., 2007). A more recent study employing aMCI patients and fMRI during resting state reported diminished anterior DMN symmetry due to increased left frontal activation (Qi et al., 2010). The results derived from the proposed local characteristic analysis (strength of healthy hubs and additional hub formation in the pathological groups) are in line with previous findings, thereby implying a reorganization of the brain's architecture during early neurodegeneartion. The additional hubs are mainly evident in the preclinical (aMCI) phase and attenuate during the onset of the clinical AD phase. This temporal pattern seems to enhance the compensatory hypothesis.

The current piece of research employed brain network analysis on EEG recordings. Despite its excellent temporal resolution, which facilitates the understanding of functional interactions among distant brain regions, EEG's spatial resolution is extremely low in comparison to other recording modalities (fMRI, PET, MEG). Moreover, it faces the problem of volume conduction especially, when analysis is not performed on the source level. Therefore, the interpretation of results, especially in the case of local characteristics analysis, should be supported by neuroimaging studies (Hämäläinen et al., 2007; Qi et al., 2010). To this extent, any synergy between EEG and fMRI with simultaneous recordings may be able to reliably track transient network alterations and their locations. However, the analysis performed herein with regards to estimating the brain network reorganization and the quantification of the underlying compensatory mechanisms was based on the definition of a Region of Interest (ROI). The ROI identification was based on the previous analysis step (identification of functional hubs) and on a priori hypothesis of an increased frontal symmetry (Hämäläinen et al., 2007; Qi et al., 2010). Despite the positive results and validating previous neuroimaging evidence (Qi et al., 2010), this point may be regarded as a current limitation, since it introduces a methodological bias posed by the study hypothesis. Finally, estimation of the disease progression was performed by forming three separate groups of participants and analyzing their brain network characteristics. However, longitudinal studies employing the same participants and investigating their network alterations during different temporal phases may estimate the disease progression much more accurately.

To sum up, this piece of work proposed a mathematical model consisting of both wavelet and brain network analysis to study neuropathological alterations due to AD and the disease progression. It provided evidence that AD evolution from its preclinical phase (aMCI) to the dementia phase is accompanied by a gradual loss of optimal brain network organization as quantified by the small-world property. This mainly occurs due to the reductions of local information processing, as expressed by lower values of the mean cluster coefficient. The degree of non-economical wiring was correlated with the amount of cognitive decline as estimated by generic neuropsychological testing (MMSE and MoCA). The functional disorganization of the EEG-based brain network is apparent during the aMCI phase. It often coexists with compensatory mechanisms involving the formation of additional hubs located mainly on left frontal and parietal regions. However, these mechanisms are transient and attenuate when progressing to the clinical AD phase. Then, the global brain network characteristics (small-world property and cluster coefficient) deteriorate much more. This computational framework seems to be a robust and reliable tool, which may be used toward the identification of functional alterations preceding structural isolation/atrophy in senior citizens facing increased risk of future progression to the clinical AD phase.

### Conflict of interest statement

The authors declare that the research was conducted in the absence of any commercial or financial relationships that could be construed as a potential conflict of interest.

## Footnotes

^1^Notice: to correct for multiple analyses we adopted a stricter significance criterion, *p* = 0.01 ^*^Bootstrapping involves the resampling of an observed dataset by randomly sampling it with replacement.

## References

[B1] Albert M. S. DeKosky S. T. Dickson D. Dubois B. Feldman H. H. Fox N. C. (2011). The diagnosis of mild cognitive impairment due to Alzheimer's Disease: recommendations from the national institute on Aging-Alzheimer's association workgroups on diagnostic guidelines for Alzheimer's Disease. Alzheimers Dement. 7, 270–279 10.1016/j.jalz.2011.03.008 21514249 PMC3312027

[B4] Bamidis P. D. Konstantinidis E. I. Billis A. Frantzidis C. Tsolaki M. Hlauschek W. (2011). A Web services-based exergaming platform for senior citizens: the long lasting memories project approach to e-health care, in Proceedings of the Annual International Conference of the IEEE Engineering in Medicine and Biology Society, EMBS, Art. No. 6090694 (Boston, MA), 2505–2509 10.1109/IEMBS.2011.6090694 22254850

[B5] Bamidis P. D. Baker N. Franco M. Losada R. Papageorgiou S. Pattichis C. S. (2012). Long Lasting Memories Project Deliverable D1.4 Final Report. Available online at: http://www.longlastingmemories.eu/sites/default/files/LLM_D1.4_final_report_public_v2.2doc.pdf, July 2012.

[B6] Bassett D. S. Bullmore E. D. (2006). Small-world brain networks. Neuroscientist 12, 512–523 10.1177/1073858406293182 17079517

[B7] Buerger K. Ewers M. Pirttilä T. Zinkowski R. Alafuzoff I. Teipel S. J. (2006). CSF phosphorylated tau protein correlates with neocortical neurofibrillary pathology in Alzheimer's Disease. Brain 129, 3035–3041 10.1093/brain/awl269 17012293

[B8] Buldú J. M. Bajo R. Maestú F. Castellanos N. Leyva I. Gil P. (2011). Reorganization of functional networks in mild cognitive impairment. PLoS ONE 6:e19584 10.1371/journal.pone.0019584 21625430 PMC3100302

[B9] Bullmore E. Sporns O. (2009). Complex brain networks: graph theoretical analysis of structural and functional systems. Nat. Rev. Neurosci. 10, 186–198 10.1038/nrn2575 19190637

[B10] Cabeza R. Anderson N. D. Locantore J. K. McIntosh A. R. (2002). Aging gracefully: compensatory brain activity in high-performing older adults. Neuroimage 17, 1394–1402 10.1006/nimg.2002.1280 12414279

[B11] Caselli R. J. Reiman E. M. Osborne D. Hentz J. G. Baxter L. C. Hernandez J. L. (2004). Longitudinal changes in cognition and behavior in asymptomatic carriers of the APOE e4 allele. Neurology 62 1990–1995 10.1212/01.WNL.0000129533.26544.BF 15184602

[B13] Citron M. (2010). Alzheimer's Disease: strategies for disease modification. Nat. Rev. Drug Discov. 9, 387–398 10.1038/nrd2896 20431570

[B14] De Haan W. Pijnenburg Y. A. Strijers R. L. Van Der Made Y. Van Der Flier W. M. Scheltens P. (2009). Functional neural network analysis in frontotemporal dementia and Alzheimer's Disease using EEG and graph theory. BMC Neurosci. 10:101 10.1186/1471-2202-10-101 19698093 PMC2736175

[B15] De Haan W. Van Der Flier W. M. Koene T. Smits L. L. Scheltens P. Stam C. J. (2012). Disrupted modular brain dynamics reflect cognitive dysfunction in Alzheimer's Disease. Neuroimage 59, 3085–3093 10.1016/j.neuroimage.2011.11.055 22154957

[B16] Delbeuck X. Van der Linden M. Collette F. (2003). Alzheimer's Disease as a disconnection syndrome? Neuropsychol. Rev. 13, 79–92 10.1023/A:1023832305702 12887040

[B17] Delorme A. Makeig S. (2004). EEGLAB: an open source toolbox for analysis of single-trial EEG dynamics including independent component analysis. J. Neurosci. Methods 134, 9–21 10.1016/j.jneumeth.2003.10.009 15102499

[B18] Drzezga A. Becker J. A. Van Dijk K. R. Sreenivasan A. Talukdar T. Sullivan C. (2011). Neuronal dysfunction and disconnection of cortical hubs in non-demented subjects with elevated amyloid burden. Brain 134, 1635–1646 10.1093/brain/awr066 21490054 PMC3102239

[B19] Dubois B. Albert M. L. (2004). Amnestic MCI or prodromal Alzheimer's Disease? Lancet Neurol. 3, 246–248 10.1016/S1474-4422(04)00710-0 15039037

[B20] Frantzidis C. A. Bratsas C. Papadelis C. L. Konstantinidis E. Pappas C. Bamidis P. D. (2010). Toward emotion aware computing: an integrated approach using multichannel neurophysiological recordings and affective visual stimuli. IEEE Trans. Inf. Technol. Biomed. 14, 589–597 10.1109/TITB.2010.2041553 20172835

[B21] Frantzidis C. A. Ladas A.-K. I. Vivas A. B. Tsolaki M. Bamidis P. D. (2014). Cognitive and physical training for the elderly: evaluating outcome efficacy by means of neurophysiological synchronization. Int. J. Psychophysiol. 93, 1–11 10.1016/j.ijpsycho.2014.01.007 24472698

[B22] González-Palau F. Franco M. Bamidis P. D. Losada R. Parra E. Papageorgiou S. (2014). The effects of a computer-based cognitive and physical training program in a healthy and mildly cognitive impaired aging sample. Aging Ment. Health. 18, 838–846 10.1080/13607863.2014.899972 24697325

[B23] Gudmundsson S. Runarsson T. P. Sigurdsson S. Eiriksdottir G. Johnsen K. (2007). Reliability of quantitative EEG features. Clin. Neurophysiol. 118, 2162–2171 10.1016/j.clinph.2007.06.018 17765604

[B24] Hämäläinen A. Pihlajamäki M. Tanila H. Hänninen T. Niskanen E. Tervo S. (2007). Increased fMRI responses during encoding in mild cognitive impairment. Neurobiol. Aging 28, 1889–1903 10.1016/j.neurobiolaging.2006.08.008 16997428

[B25] He Y. Chen Z. Evans A. (2008). Structural insights into aberrant topological patterns of large-scale cortical networks in Alzheimer's Disease. J. Neurosci. 28, 4756–4766 10.1523/JNEUROSCI.0141-08.2008 18448652 PMC6670444

[B26] Hsu Y. F. Huang Y. Z. Lin Y. Y. Tang C. W. Liao K. K. Lee P. L. (2012). Intermittent theta burst stimulation over ipsilesional primary motor cortex of subacute ischemic stroke patients: a pilot study. Brain Stimul. 6, 166–174 10.1016/j.brs.2012.04.007 22659021

[B28] Lithari C. Klados M. A. Pappas C. Albani M. Kapoukranidou D. Kovatsi L. (2012). Alcohol Affects the Brain's Resting-State Network in Social Drinkers. PLoS ONE 7:e48641 10.1371/journal.pone.0048641 23119078 PMC3485329

[B29] McKhann G. Drachman D. Folstein M. Katzman R. Price D. Stadlan E. M. (1984). Clinical diagnosis of Alzheimer's Disease report of the NINCDS−ADRDA work group^*^ under the auspices of department of health and human services task force on Alzheimer's Disease. Neurology 34, 939–939 10.1212/WNL.34.7.939 6610841

[B30] Mitchell A. J. Shiri-Feshki M. (2009). Rate of progression of mild cognitive impairment to dementia–meta-analysis of 41 robust inception cohort studies. Acta Psychiatr. Scand. 119, 252–265 10.1111/j.1600-0447.2008.01326.x 19236314

[B31] Moretti D. V. D. Paternic, ò, Binetti G. Zanetti O. Frisoni G. B. (2013). EEG upper/low alpha frequency power ratio relates to temporo-parietal brain atrophy and memory performances in mild cognitive impairment. Front. Aging Neurosci. 5:63 10.3389/fnagi.2013.00063 24187540 PMC3807715

[B32] Moretti D. V. Frisoni G. B. Fracassi C. Pievani M. Geroldi C. Binetti G. (2011). MCI patients' EEGs show group differences between those who progress and those who do not progress to AD. Neurobiol. Aging 32, 563–571 10.1016/j.neurobiolaging.2009.04.003 20022139

[B33] Moretti D. V. Prestia A. Fracassi C. Binetti G. Zanetti O. Frisoni G. B. (2012). Specific EEG changes associated with atrophy of hippocampus in subjects with mild cognitive impairment and Alzheimer's Disease. Int. J. Alzheimers Dis. 2012:253153 10.1155/2012/253153 22506130 PMC3296269

[B34] Petersen R. C. (2004). Mild cognitive impairment as a diagnostic entity. J. Intern. Med. 256, 183–194 10.1111/j.1365-2796.2004.01388.x 15324362

[B36] Petersen R. C. Smith G. E. Waring S. C. Ivnik R. J. Tangalos E. G. Kokmen E. (1999). Mild cognitive impairment: clinical characterization and outcome. Arch. Neurol. 56, 303 10.1001/archneur.56.3.303 10190820

[B37] Qi Z. Wu X. Wang Z. Zhang N. Dong H. Yao L. (2010). Impairment and compensation coexist in amnestic MCI default mode network. Neuroimage 50, 48–55 10.1016/j.neuroimage.2009.12.025 20006713

[B38] Quian Quiroga R. Schürmann M. (1999). Functions and sources of event-related EEG alpha oscillations studied with the wavelet transform. Clin. Neurophysiol. 110, 643–654 10.1016/S1388-2457(99)00011-5 10378733

[B39] Rosso O. A. Blanco S. Yordanova J. Kolev V. Figliola A. Schurmann M. (2001). Wavelet entropy: a new tool for analysis of short duration brain electrical signals. J. Neurosci. Methods 105, 65–76 10.1016/S0165-0270(00)00356-3 11166367

[B40] Sanz-Arigita E. J. Schoonheim M. M. Damoiseaux J. S. Rombouts S. A. Maris E. Barkhof F. (2010). Loss of ‘small-world’networks in Alzheimer's Disease: graph analysis of FMRI resting-state functional connectivity. PLoS ONE 5:e13788 10.1371/journal.pone.0013788 21072180 PMC2967467

[B41] Seo E. H. Lee D. Y. Lee J. M. Park J. S. Sohn B. K. Lee D. S. (2013). Whole-brain functional networks in cognitively normal, mild cognitive impairment, and Alzheimer's Disease. PLoS ONE 8:e53922 10.1371/journal.pone.0053922 23335980 PMC3545923

[B42] Sperling R. A. Aisen P. S. Beckett L. A. Bennett D. A. Craft S. Fagan A. M. (2011). Toward defining the preclinical stages of Alzheimer's Disease: recommendations from the national institute on Aging-Alzheimer's association workgroups on diagnostic guidelines for Alzheimer's Disease. Alzheimers Dement. 7, 280–292 10.1016/j.jalz.2011.03.003 21514248 PMC3220946

[B43] Stam C. J. De Haan W. Daffertshofer A. Jones B. F. Manshanden I. van Walsum A. V. C. (2009). Graph theoretical analysis of magnetoencephalographic functional connectivity in Alzheimer's Disease. Brain 132, 213–224 10.1093/brain/awn262 18952674

[B44] Stam C. J. Jones B. F. Nolte G. Breakspear M. Scheltens P. (2007). Small-world networks and functional connectivity in Alzheimer's Disease. Cereb. Cortex 17, 92–99 10.1093/cercor/bhj127 16452642

[B45] Stam C. J. Reijneveld J. C. (2007). Graph theoretical analysis of complex networks in the brain. Nonlinear Biomed. Phys. 1:3 10.1186/1753-4631-1-3 17908336 PMC1976403

[B46] Supekar K. Menon V. Rubin D. Musen M. Greicius M. D. (2008). Network analysis of intrinsic functional brain connectivity in Alzheimer's Disease. PLoS Comput. Biol. 4:e1000100 10.1371/journal.pcbi.1000100 18584043 PMC2435273

[B48] Unser M. Akram A. Murray E. (1992). On the asymptotic convergence of B-spline wavelets to Gabor functions. IEEE Trans. Inf. Theory 38, 864–872 10.1109/18.119742

[B50] Van Dijk K. R. Hedden T. Venkataraman A. Evans K. C. Lazar S. W. Buckner R. L. (2010). Intrinsic functional connectivity as a tool for human connectomics: theory, properties, and optimization. J. Neurophysiol. 103, 297–321 10.1152/jn.00783.2009 19889849 PMC2807224

[B51] Vos S. J. van Rossum I. A. Verhey F. Knol D. L. Soininen H. Wahlund L. O. (2013). Prediction of Alzheimer disease in subjects with amnestic and nonamnestic MCI. Neurology 80, 1124–1132 10.1212/WNL.0b013e318288690c 23446677

[B52] Wang J. Zuo X. Dai Z. Xia M. Zhao Z. Zhao X. (2013). Disrupted functional brain connectome in individuals at risk for Alzheimer's Disease. Biol. Psychiatry 73, 472–481 10.1016/j.biopsych.2012.03.026 22537793

[B53] Watts D. J. Strogatz S. H. (1998). Collective dynamics of ‘small-world’networks. Nature 393, 440–442 10.1038/30918 9623998

[B54] Yao Z. Zhang Y. Lin L. Zhou Y. Xu C. Jiang T. (2010). Abnormal cortical networks in mild cognitive impairment and Alzheimer's Disease. PLoS Computat. Biol. 6:e1001006 10.1371/journal.pcbi.1001006 21124954 PMC2987916

[B55] Zhao X. Liu Y. Wang X. Liu B. Xi Q. Guo Q. (2012). Disrupted small-world brain networks in moderate Alzheimer's Disease: a resting-state FMRI study. PLoS ONE 7:e33540 10.1371/journal.pone.0033540 22457774 PMC3311642

