# Supplementary material for: Functional disorganization of small-world brain networks in mild Alzheimer's Disease and amnestic Mild Cognitive Impairment: an EEG study using Relative Wavelet Entropy (RWE)
Source: Front Aging Neurosci. 2014 Aug 26;6:224. doi: 10.3389/fnagi.2014.00224 (PMC4144118; doi:10.3389/fnagi.2014.00224)
Supplement: Supplementary file 1 [file DataSheet1.DOCX]

1. **Appendix**

Table A1: Small world mean values (with ± standard deviations) as a function of density (number of edges), for each of the three groups (Healthy, aMCI, MD) and for each density range (500, 600, 700, 800 edges). The number of participants per group and standard errors are also displayed.

| **Group** | **Number of Edges (N)** | **Number of Participants** | **Small world (Mean±Std.Dev)** | **Std. Error** |
| --- | --- | --- | --- | --- |
| Healthy | 500 | 23 | 2.6775 ± 0.1044 | 0.0218 |
| aMCI |  | 17 | 2.5976 ± 0.1213 | 0.0294 |
| MD |  | 24 | 2.6099 ± 0.1218 | 0.0223 |
| Healthy | 600 | 23 | 2.3125 ± 0.0889 | 0.0185 |
| aMCI |  | 17 | 2.2356 ± 0.0813 | 0.0197 |
| MD |  | 24 | 2.1866 ± 0.0627 | 0.0128 |
| Healthy | 700 | 23 | 2.0418 ± 0.0572 | 0.0119 |
| aMCI |  | 17 | 1.9709 ± 0.0657 | 0.0159 |
| MD |  | 24 | 1.9191 ± 0.0508 | 0.0104 |
| Healthy | 800 | 23 | 1.8359 ± 0.0464 | 0.0097 |
| aMCI |  | 17 | 1.7812 ± 0.0635 | 0.0154 |
| MD |  | 24 | 1.7352 ± 0.0416 | 0.0085 |

Table A2: Cluster Coefficient mean values (with ± standard deviations) as a function of density (number of edges), described for each of the three groups (Healthy, aMCI, MD) and for each density range (500, 600, 700, 800 edges). The number of participants per group and the standard errors are also reported.

| **Group** | **Number of Edges (N)** | **Number of Participants** | **Cluster Coefficient (Mean±Std.Dev)** | **Std. Error** |
| --- | --- | --- | --- | --- |
| Healthy | 500 | 23 | 0.5133 ± 0.0382 | 0.0080 |
| aMCI |  | 17 | 0.4851 ± 0.0349 | 0.0085 |
| MD |  | 24 | 0.4684 ± 0.0231 | 0.0047 |
| Healthy | 600 | 23 | 0.5420 ± 0.0352 | 0.0073 |
| aMCI |  | 17 | 0.5128 ± 0.0332 | 0.0080 |
| MD |  | 24 | 0.4985 ± 0.0272 | 0.0056 |
| Healthy | 700 | 23 | 0.5628 ± 0.0325 | 0.0068 |
| aMCI |  | 17 | 0.5335 ± 0.0327 | 0.0079 |
| MD |  | 24 | 0.5214 ± 0.0322 | 0.0066 |
| Healthy | 800 | 23 | 0.5793 ± 0.0293 | 0.0061 |
| aMCI |  | 17 | 0.5536 ± 0.0339 | 0.0082 |
| MD |  | 24 | 0.5426 ± 0.0350 | 0.0072 |

Table A3: Characteristic Path Length mean values (with ± standard deviation) as a function of density, for each of the three groups (Healthy, aMCI, MD) and for each density range (500, 600, 700, 800 edges). The number of participants per group and standard errors are also reported.

| **Group** | **Number of Edges**  **(N)** | **Number of Participants** | **Characteristic Path Length (Mean±Std.Dev)** | **Std. Error** |
| --- | --- | --- | --- | --- |
| Healthy | 500 | 23 | 2.4115 ± 0.1917 | 0.0080 |
| aMCI |  | 17 | 2.3456 ± 0.1313 | 0.0085 |
| MD |  | 24 | 2.3145 ± 0.1412 | 0.0047 |
| Healthy | 600 | 23 | 2.2336 ± 0.1353 | 0.0073 |
| aMCI |  | 17 | 2.1873 ± 0.0910 | 0.0080 |
| MD |  | 24 | 2.1767 ± 0.1005 | 0.0056 |
| Healthy | 700 | 23 | 2.0924 ± 0.0937 | 0.0068 |
| aMCI |  | 17 | 2.0564 ± 0.0651 | 0.0079 |
| MD |  | 24 | 2.0661 ± 0.1153 | 0.0066 |
| Healthy | 800 | 23 | 1.9783 ± 0.0712 | 0.0061 |
| aMCI |  | 17 | 1.9484 ± 0.0557 | 0.0082 |
| MD |  | 24 | 1.9622 ± 0.1090 | 0.0072 |
